# Astrocyte transplantation for repairing the injured spinal cord

**DOI:** 10.7555/JBR.36.20220012

**Published:** 2022-06-28

**Authors:** Xiaolong Zheng, Wei Wang

**Affiliations:** 1 Department of Neurology, Tongji Hospital, Tongji Medical College, Huazhong University of Science and Technology, Wuhan, Hubei 430030, China; 2 Key Laboratory of Neurological Diseases of Chinese Ministry of Education, the School of Basic Medicine, Tongji Medical College, Huazhong University of Science and Technology, Wuhan, Hubei 430030, China

**Keywords:** spinal cord injury, astrocyte, transplantation, regeneration

## Abstract

Spinal cord injury (SCI) leads to permanent deficits in neural function without effective therapies, which places a substantial burden on families and society. Astrocytes, the major glia supporting the normal function of neurons in the spinal cord, become active and form glial scars after SCI, which has long been regarded as a barrier for axon regeneration. However, recent progress has indicated the beneficial role of astrocytes in spinal repair. During the past three decades, astrocyte transplantation for SCI treatment has gained increasing attention. In this review, we first summarize the progress of using rodent astrocytes as the primary step for spinal repair. Rodent astrocytes can survive well, migrate extensively, and mature in spinal injury; they can also inhibit host reactive glial scar formation, stimulate host axon regeneration, and promote motor, sensory, respiratory, and autonomic functional recovery. Then, we review the progress in spinal repair by using human astrocytes of various origins, including the fetal brain, fetal spinal cord, and pluripotent stem cells. Finally, we introduce some key questions that merit further research in the future, including rapid generation of large amounts of human astrocytes with high purity, identification of the right origins of astrocytes to maximize neural function improvement while minimizing side effects, testing human astrocyte transplantation in chronic SCI, and verification of the long-term efficacy and safety in large animal models.

## Introduction

Traumatic spinal cord injury (SCI) leads to permanent neurological deficits in motor, sensory and autonomic function that cannot be cured by current treatments^[1]^, which places a substantial burden on individuals, families and society^[2]^. Histologically, the spinal cord is composed of many cells and structures, including neurons, glia, axons and myelin, all of which are lost in the injury epicenter after the spinal cord damage, producing a large cavity^[3]^. Thus, transplanting exogenous cells to replace the lost cells is a promising strategy to repair the injured spinal cord. In the past 30 years, many kinds of cells have been experimentally transplanted to treat SCI^[4–5]^, including Schwann cells, olfactory ensheathing cells (OECs), mesenchymal stromal cells (MSCs), neural progenitor cells (NPCs), and oligodendrocyte progenitor cells (OPCs). These grafted cells partially promote functional recovery by several mechanisms, such as neuroprotection, immunomodulation, axon regeneration and sprouting, relay formation and remyelination^[4–5]^. Of note, NPCs and OPCs are receiving superior attention to other cells for transplantation in that NPCs produce neurons that can form relay circuits^[6]^, and OPCs produce oligodendrocytes that can remyelinate axons^[7–9]^, both of which are unable to regenerate spontaneously after SCI. However, there are still challenges limiting the effect of grafting NPCs and OPCs for treating SCI, including poor graft survival, lack of functional synaptic connections, and myelin formation.

Astrocytes are abundant glia in the spinal cord that structurally and functionally support the normal function of neurons^[10]^. After the spinal cord is injured, astrocytes proliferate and become active, forming extensive scars and secreting extracellular matrix surrounding the lesion. Reactive astrocytes have long been regarded as a barrier for axonal regeneration after SCI^[11]^; however, recent progress has uncovered the beneficial role of astrocytes in repairing lesioned spinal cords. For example, the abolishment of reactive astrocytes after spinal crush injury in mice causes widespread tissue disruption, pronounced cellular degeneration, and failure of wound contraction, with severe persisting motor deficits^[12–13]^. Moreover, spontaneous regrowth of transected corticospinal, sensory, or serotonergic axons through severe SCI lesions failed after preventing reactive astrocyte formation^[14]^. Thus, astrocytes could be manipulated to reshape the microenvironment of the lesion to harness benefits in the repair of injured spinal cord. In addition, astrocytes could be added to neuron grafts^[15]^ since astrocytes could support neuron maturation^[16]^ and enhance synaptic plasticity^[17]^. Astrocytes also enhance oligodendrogenesis or remyelination^[18]^. In the past three decades, studies on astrocyte transplantation for SCI treatment have been carried out and the progress has been summarized in several excellent reviews^[19–24]^. However, the proper origins of astrocytes for SCI treatment remain unclear. Here, we reintroduce this field from a different perspective with the aim to maximize the beneficial effect of astrocyte transplantation in spinal cord repair.

## Employing rodent astrocytes as a first step for spinal cord injury treatment

**Figure 1:**
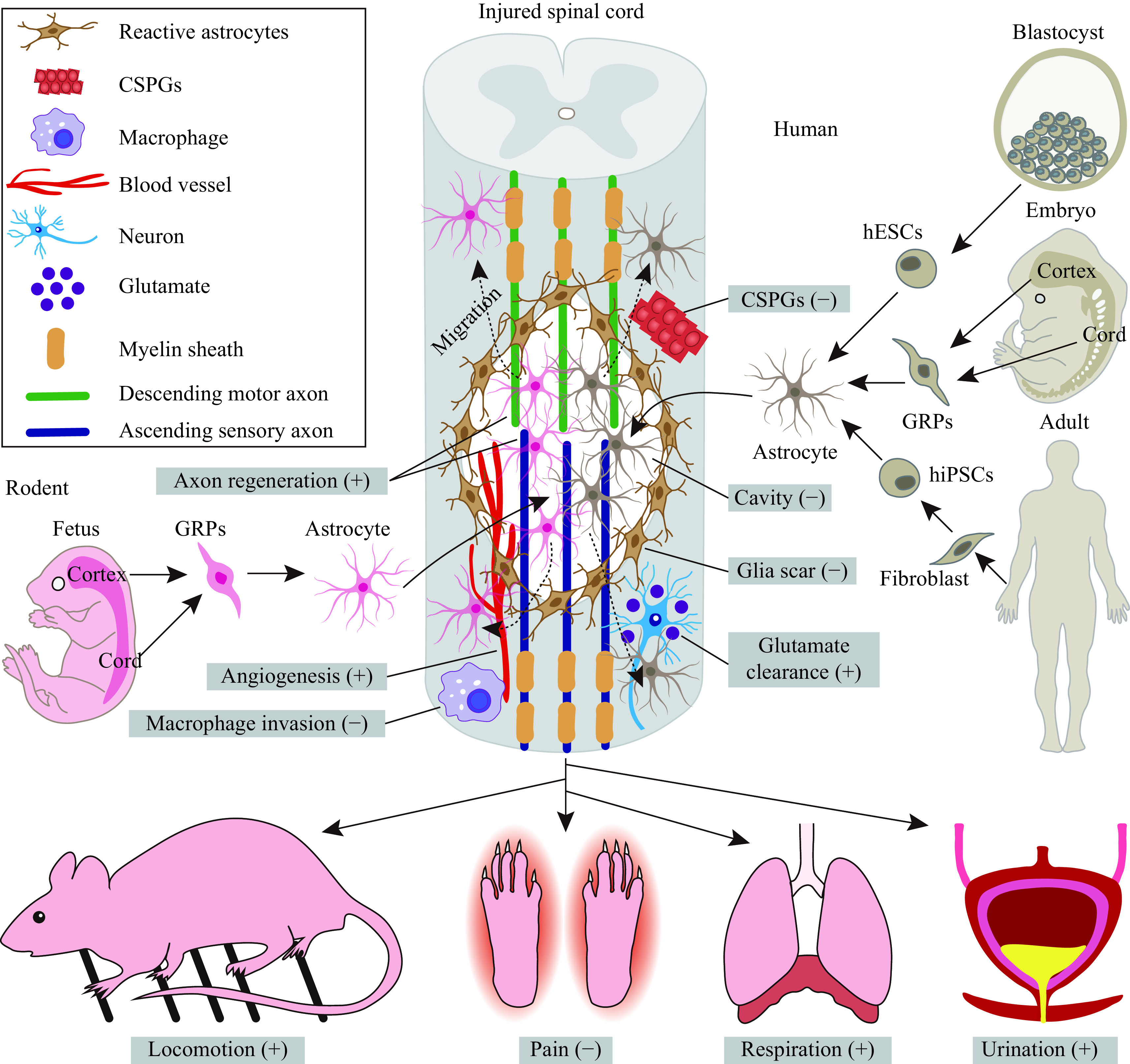
Transplantation of astrocytes to repair the injured spinal cord.

Because of their easy availability and rapid maturation, rodent astrocytes were initially transplanted to test their efficacy in repairing spinal lesions and promoting functional recovery (***Fig. 1***). These grafted rodent astrocytes are primarily derived from both the embryonic and postnatal brain and spinal cord (***Fig. 1***), which means that they are in fact immature progenitors in development. When grafted into the lesioned spinal cord, these cells survive, migrate and mature, promoting motor, sensory, respiratory and autonomic functional recovery (***Fig. 1***).

### Fate of grafted rodent astrocytes

The key to cell transplantation in SCI is the survival and differentiation of grafted cells. When glial restricted progenitors (GRPs) derived from the embryonic rat spinal cord are grafted into the naïve spinal cord, these cells survive well for at least 6 weeks, differentiate into nearly pure astrocytes, and migrate extensively in the white matter both rostrally and caudally^[25]^. Based on this evidence, these GRPs are again grafted into lateral funiculus transection lesions and survive the lesion environment for at least 5 weeks, having the same differentiation and migration pattern as when grafted into the naïve spinal cord^[25]^. Furthermore, when neuronal restricted progenitors (NRPs) are added to GRP grafts, they even completely fill the lesion^[26]^; when these GRPs are pretreated with different factors, all of them show equivalent survival and differentiation^[27]^. However, when the same GRPs are grafted into the contusion injury, they only survive within the parenchyma around the cavity, and no cells present within the cavity^[28]^. In addition to spinal astrocytes, cortical astrocytes derived from both postnatal^[29]^ and adult^[30]^ rats can also survive and migrate when grafted into spinal hemisection injury. In summary, astrocytes with different origins can survive a variety of spinal injuries for a relatively long period, paving the way for their potential in SCI treatment.

### Host reaction to grafted rodent astrocytes

The notion for grafting astrocytes to cure SCI is to promote axon regrowth. Initially, embryonic rat spinal cord-derived GRPs have been found to stimulate the regrowth of crushed dorsal root axons into the dorsal column and gray matter^[31]^, and they can also induce regrowth of the transected ascending dorsal column sensory axons into grafts^[27]^; however, these axons never grow out beyond the transected lesion. For the descending motor axons, the corticospinal tract (CST) and the raphespinal tract seem to contact these GRPs grafted into contusion lesions; however, these tracts still cannot regenerate out of the lesion cavity^[28]^. In fact, CST fails to regrow into the grafted GRPs^[32]^. In addition to spinal GRPs, neocortical astrocytes can also stimulate the growth of lesioned axons into grafts^[29,33–34]^; yet, the kinds of axons that grow remain unclear. Besides regeneration, the grafted astrocytes exert other effects on the injured host spinal cord; they can inhibit host reactive scar formation^[28–29]^ and reduce the lesion cavity^[29]^. In addition, macrophage infiltration is alleviated^[28]^, secretion of axon growth inhibiting extracellular matrix is reduced^[28]^, and angiogenesis is enhanced^[33]^. All these reactions can partly explain the beneficial effects of transplanting astrocytes for treating SCI.

### Promotion of motor recovery

Regaining motor control is essential for the SCI population to achieve primary functional recovery^[35–36]^. Initially, grafting embryonic spinal astrocytes into dorsal column transection significantly worsens hindlimb fine motor control^[37]^. In addition, grafted embryonic spinal GRPs^[38]^ or GRP-derived astrocytes with ciliary neurotrophic factor (CNTF) differentiation (GDAs^CNTF^)^[39]^ deposit many chondroitin sulfate proteoglycans (CSPGs), preventing the growth of both dorsal column axons and the rubrospinal tract, and failing to improve fine motor control^[38–39]^. However, when grafting GRP-derived astrocytes with bone morphogenetic protein (BMP) differentiation (GDAs^BMP^), surprisingly, the dorsal column axons and rubrospinal tract can robustly regenerate into grafts and beyond the lesion, and fine motor control is significantly improved^[38]^, probably due to less secreted CSPGs and realignment of host astrocyte processes in parallel enabling axon regeneration^[38]^. Unfortunately, neither GRPs nor GDAs^BMP^ could preserve host spinal tissue or improve hindlimb locomotion when grafted subacutely after contusion lesion^[40]^, but once immediately grafted after injury, GDAs^BMP^ could preserve more corticospinal and sensory axons and improve hindlimb locomotion^[41]^. Additionally, GDAs^BMP^ engineered to express multineurotrophin D15A^[40]^ or supplemented with human recombinant decorin^[41]^ can significantly increase spinal tissue preservation and hindlimb locomotion. Moreover, when GRPs are mixed into NRPs and cografted, axons, including 5-hydroxytryptamine (5-HT) fibers, can regenerate with significantly elevated hindlimb general locomotion^[42]^; however, improved fine motor control of hindlimbs cannot be observed^[42]^. In addition to GRPs, neocortical astrocytes can promote the growth of many axons after dorsal hemisection^[43]^ or compression^[44]^, and improve general locomotion but fail to enhance fine motor control.

### Influence on sensory function

Sensory function, particularly neuropathic pain after SCI, is often neglected in cell transplantation research. This is very important since grafted cells can aggravate pain^[45–46]^. In fact, grafting of GRPs or GDAs^CNTF^ leads to both mechanical and thermal allodynia as early as 3 weeks post dorsal column transection^[39]^; in contrast, allodynia does not develop in GDAs^BMP^ grafts^[39]^, while significantly increased aberrant sprouting of calcitonin gene-related peptide (CGRP) in the dorsal horn is only present in GRPs or GDAs^CNTF^ grafts but not in GDAs^BMP^ grafts^[39]^. Interestingly, when GRPs or GDAs^BMP^ are grafted into contusion injury, mechanical allodynia does not develop^[40]^. Moreover, when GRPs are mixed into NRPs and cografted, thermal hypersensitivity is diminished as early as one-week post grafting, and the sprouting of CGRP fibers is inhibited in the dorsal horn^[42]^. Grafting of mouse-induced pluripotent stem cells (iPSCs) differentiated astrocytes into contusion lesions can induce mechanical but not thermal allodynia; however, aberrant CGRP fiber sprouting does not increase in the dorsal horn^[47]^. In addition, grafting neocortical astrocytes fails to cause any apparent signs of increased pain sensitivity or the presence of chronic pain or self-mutilation^[44]^.

### Promotion of respiratory function

SCI often occurs in the cervical spine, causing breath distress, and some studies have tried to find treatments, including astrocyte transplantation. Grafting GRPs into cervical hemisection injury not only stimulates robust regeneration of the ipsilateral bulbospinal respiratory tract and 5-HT fibers but also induces sprouting of the contralateral bulbospinal respiratory tract. Electromyogram recordings from the ipsilateral hemidiaphragm revealed significantly increased burst amplitudes, demonstrating substantial recovery of diaphragm function^[48]^. However, when GDAs^BMP^ are grafted into cervical hemicontusion injury, no improvement in diaphragm function is observed^[49]^; if GDAs^BMP^ are engineered to overexpress glutamate transporter 1 (GLT-1) to enhance glutamate uptake, the lesion volume, number of phrenic motor neurons and diaphragm innervation are rescued, and diaphragm function is improved^[49]^.

### Promotion of autonomic function

SCI also leads to autonomic dysfunction, including bladder dysfunction, which severely affects the quality of life; however, few studies have attempted to address this problem with astrocyte transplantation. When GRPs are mixed with NRPs and cografted into contusion injury, accelerated recovery of bladder contraction from the spinal shock phase and increased voiding efficiency are observed as early as 2 weeks post grafting. At 8 weeks after transplantation, urodynamic parameters, including micturition pressure, residual urine, bladder capacity, and bladder weight, are reduced^[42]^.

## Moving into human astrocytes in preclinical translation studies

Though rodent astrocyte transplantation shows promising beneficial results in animal models of SCI, it cannot be used to treat human SCI because of xenotransplantation. Instead, human astrocytes should be grafted; however, preclinical research should be done before they enter clinical practice. To date, human astrocytes from several origins have shown promise in treating SCI (***Fig. 1***).

### Human fetal brain-derived astrocytes

While the most commonly used rodent astrocytes in spinal repair are of fetal spinal cord origin, the more studied human fetal astrocytes for treating SCI were obtained from the brain. Direct transplantation of human fetal brain GRPs in spinal hemisection sites can induce regeneration of both the ascending dorsal column sensory axons and descending motor axons, including reticulospinal and raphespinal tracts, while other descending motor axons, such as coerulospinal and rubrospinal tracts, did not show any increased regrowth^[50]^. Further differentiation of human fetal brain GRPs into astrocytes of different maturities with BMP or CNTF also results in equal survival in dorsal column transection lesions and promoted similar growth of ascending sensory axons^[51]^. However, this is not the case in spinal contusion injury. Although direct grafting of human fetal brain GRPs into the contusion epicenter can reduce the lesion cavity, suppress both glial and fibrotic scar formation and aid in the regrowth of 5-HT fibers, general locomotion, fine motor control, and sensory and bladder function cannot show significant improvement^[52]^. In contrast, when these human fetal brain GRPs are predifferentiated into astrocytes by BMP, sensory and bladder function recover significantly, although general locomotion and fine motor control are still not improved^[52]^.

### Human fetal spinal cord-derived astrocytes

The role of human fetal astrocytes (from the fetal spinal cord) in SCI treatment has only been reported in one study^[53]^. Directly grafting human fetal spinal GRPs or their derived astrocytes with CNTF differentiation after hemisection lesion can neither protect host spinal neurons from death nor improve fine motor control^[53]^; in contrast, only BMP-induced astrocytes can stimulate robust axon regeneration and promote host spinal neurons from death, thus significantly improving fine motor control as early as one-week post grafting^[53]^.

### Human embryonic stem cell-differentiated astrocytes

The derivation of human embryonic stem cells (hESCs)^[54–56]^ enables the differentiation of all kinds of cells in adult humans. Due to the establishment of a protocol for differentiating hESCs into neural progenitors^[57–58]^, astrocytes can also be obtained from hESCs. Initially, grafted hESC-derived cerebral neural progenitors profoundly differentiate into mature astrocytes in the naïve spinal cord, migrating extensively and integrating structurally within the host spinal cord over 9 months^[59]^. When grafted into spinal hemisection lesions, nearly half of hESC-derived cerebral neural progenitors become astrocytes after 18 months, which extensively migrate both rostrally and caudally^[60]^. Moreover, these differentiated astrocytes suppress host reactive glial scar formation in the injury site, adopting blood–spinal cord barrier phenotypes, supporting neuronal function by regulating neurotransmitter levels, and protecting neurons from excitotoxicity^[61]^.

In addition, spinal astrocyte progenitors can be differentiated from hESCs^[62]^, and they survive well for at least 12 weeks in the naïve spinal cord, differentiating exclusively into astrocytes^[63]^. Gene profiling has revealed that these grafted human astrocytes become mature, expressing both structural and functional proteins of astrocytes, while genes related to neural progenitors, oligodendroglia, microglia, and neurons are almost absent^[63]^. However, it is unclear whether hESC-derived spinal astrocytes can aid in functional recovery after SCI, which merits further investigation.

### Human induced pluripotent stem cell differentiated astrocytes

The discovery of iPSCs enables the obtainment and autologous transplantation of any cells without immune rejection^[64]^. Specifically, the generation of human iPSCs^[65–66]^ holds great promise in clinical translation. Human spinal astrocytes with high purity can be obtained from human iPSCs^[62]^, and they can survive and mature in 12 weeks after grafting into the naïve spinal cord^[63]^. However, although they can survive and mature into astrocytes in spinal hemicontusion lesions, they fail to improve respiratory function^[67]^. This was probably due to the absence of GLT-1 expression in human iPSC-derived astrocytes, since overexpression of GLT-1 enables high glutamate uptake, significantly reducing the lesion area, preserving the innervation of the diaphragm, and improving respiratory function^[67]^.

Although transplantation of human iPSC-derived astrocytes is promising in treating neurological disorders, the potential safety concerns prevent their use in clinical practice. For example, grafting human iPSC-derived astrocytes from amyotrophic lateral sclerosis (ALS) patients into the naïve spinal cord may induce ALS-like symptoms in host mice^[59]^, and human astrocytes with ALS may induce motor neuron degeneration in host mice^[68]^. Thus, the origin of human iPSCs is vital to cell transplantation.

## Perspectives

Although recent progress in astrocyte transplantation for SCI treatment is a prospective method (***Fig. 1***), there are still many challenges before it enters clinical trials.

The first challenge is to ensure the sufficient amount of human astrocytes for transplantation, as the scale of humans is much more escalated than rodents; that is, one rat may need only a million cells, while more than hundreds of millions of cells are needed for an individual. Isolating enough astrocytes from the human fetal spinal cord or brain can hardly meet the demand, while directed differentiation of astrocytes from human ESCs or iPCSs can be a better solution because of their high pluripotency and proliferation. However, it takes almost 6 months to obtain functional astrocytes from hESCs^[69–70]^, and the differentiation time is much longer than the optimal time window for cell transplantation (usually 2 to 4 weeks after injury). Therefore, rapid generation of human functional astrocytes from human ESCs and iPSCs is needed. By inducible expression of nuclear factor IA, astrocytes can be efficiently generated in 4 to 7 weeks^[71–72]^, which tremendously accelerates the generation of functional human astrocytes in large numbers.

The second unresolved problem is whether human spinal or cerebral astrocytes, which are better for treating SCI, display diverse morphologies in different regions of the central nervous system. RNA sequencing of human pluripotent stem cell-derived regional astrocytes reveals distinct transcript profiles, suggesting differential functional properties, such as effects on neurite growth and blood-brain barrier formation^[73]^. There is evidence supporting the use of spinal NPCs rather than cerebral NPCs since only spinal NPCs can induce CST regeneration^[32,74]^. It seems that human spinal astrocytes are superior for integrating into the spinal cord and improving functional recovery; however, this needs further exploration.

In addition, in almost all current studies, astrocytes are transplanted in the acute or subacute phase after SCI, and whether human astrocytes could be used to treat chronic spinal injuries remains unclear. This is a vital problem because most individuals with spinal injuries are in the chronic phase, and patients are specifically concerned about the effects of cell transplantation on chronic SCI^[75]^. And the long-term effects of astrocyte transplantation on other complications related to spinal injury, such as spasms or repeated hypotensive episodes, need close observation and in-depth study because these syndromes may largely impair quality of life.

Finally, the efficacy and safety of transplanting astrocytes for treating SCI have only been tested in rodent animal models. Even though the rodent spinal cord shares many similarities with that of humans, there are still differences between them. For example, the length and diameter of the human spinal cord are much larger than those of the rat spinal cord. In addition, the CST is in the dorsal column of the rat spinal cord, while it is in the lateral column of the human spinal cord. The locomotion of rats is solely quadrupedal, while humans engage in bipedal walking. The lifetime of rodents is much shorter than that of humans, and therefore the observation period on rats is insufficient to recognize the potential unwanted side effects. In summary, the findings gained from rodents are insufficient to prove the safety and efficacy of astrocyte transplantation. This gap may be resolved by applying a large animal nonhuman primate SCI model that resembles human SCI both anatomically and physiologically^[76–78]^ to observe the long-term safety and efficacy of astrocyte transplantation before initiating clinical trials.

Although many basic studies on astrocyte transplantation for spinal repair have been conducted, unfortunately, no clinical trial has been initiated currently. By contrast, transplantation of other cells, such as Schwann cells, OECs, MSCs, NPCs, and OPCs, have been tested in several clinical trials. This sharp difference could partly be attributed to the notion that astrocytes can self-proliferate extensively and form reactive astrogliosis, which has long been regarded as a barrier to axon regeneration; thus, transplanting exogenous astrocytes seems unnecessary or even harmful to spinal repair, making researchers reluctant to delve into the benefits of astrocyte transplantation for SCI and thus leading to insufficient evidence for the initiation of clinical trials. Thus, more studies regarding the ideal origins of astrocytes (*e.g.*, at higher cervical lesion levels), chronic SCI phase, severe complete SCI lesions, prolonged observation periods of astrocyte transplantation, and large animal models of SCI should be conducted, which can pave the way for the clinical translation of astrocyte transplantation for SCI treatment.
